# Hendra virus genotypes 1 and 2 differ in V protein-mediated immune evasion

**DOI:** 10.1099/jgv.0.002256

**Published:** 2026-04-21

**Authors:** Melanie N. Tripp, Stephen M. Rawlinson, Sarah J. Edwards, Cassandra T. David, Glenn A. Marsh, Kim Halpin, Gregory W. Moseley

**Affiliations:** 1 Department of Microbiology, Biomedicine Discovery Institute, Monash University, Clayton, VIC 3800, Australia; 2 Australian Centre for Disease Preparedness, Commonwealth Scientific and Industrial Research Organisation (CSIRO), East Geelong, VIC 3219, Australia

**Keywords:** Hendra virus, henipavirus, immune evasion, V protein, virus–host interaction, zoonoses

## Abstract

Hendra virus (HeV) is a highly pathogenic virus endemic to Australia that causes lethal infections in horses and humans following spillover from bat reservoirs. There are two known genotypes: genotype 1 and 2 (HeV-g1 and HeV-g2). Both have caused lethal disease in horses, but HeV-g1 causes a more severe disease than HeV-g2 in non-human primates. The molecular mechanisms underlying these differences are poorly understood. The capacity of viruses to evade the interferon (IFN)-mediated antiviral innate immune response is important in infection and disease, and in HeV, the virulence factor V protein is a key mediator and one of the most divergent proteins between the genotypes. We compared the IFN antagonist functions of the V proteins of HeV-g1 and HeV-g2, finding that HeV-g1-V was more potent than HeV-g2-V in antagonizing the induction of type-I IFN; we further found that the proteins differ in nucleocytoplasmic localization. Consistent with these findings, HeV-g1 suppressed type-I IFN production more effectively than HeV-g2 during infection. These data reveal differences in the fundamental biology of HeV genotypes, which may be significant to pathogenesis.

## Introduction

Hendra virus (HeV) is a highly pathogenic paramyxovirus that, along with Nipah virus (NiV), comprises the first characterized species of the *Henipavirus* genus [1]. The genus includes three other members, all with bat reservoir hosts and variously identified in Australia, Asia and Africa [2,5]. Of these, only HeV and NiV are known to have caused human infection, which is characterized by severe respiratory disease and encephalitis with high mortality rates [1]. HeV and NiV are risk group 4 viruses with no currently approved therapeutics or vaccines for humans [1], although a HeV vaccine based on soluble G protein is approved for use in horses [6].

There are two genotypes of HeV (genotypes 1 and 2). HeV-g1 was identified in 1994 (in Hendra, Australia) and has infected >100 horses and 7 humans (causing 4 human deaths) [1]. HeV genotype 2 (HeV-g2) was identified in fruit bat surveillance in 2021, including in Australian states considered to be low risk for HeV spillover [7]. HeV-g2 has not been reported to infect humans but has caused two fatal infections in horses, one retrospectively identified from 2015 [8] and another in 2021 [9]. Research on HeV-g2 has been relatively limited, but analysis of fusion (F) and attachment (G) glycoproteins indicates conserved receptor tropism and cross-reactivity with monoclonal antibodies against HeV-g1 and NiV, suggesting that HeV/NiV G protein-based vaccines/therapeutics would be effective against HeV-g2 [10].

Recently, virus challenge experiments in African green monkeys (AGMs) identified reduced pathogenicity and mortality rates in HeV-g2-infected compared with HeV-g1-infected AGMs [11]. *In vitro* analysis indicated slower growth of HeV-g2 compared with HeV-g1, and lower levels of HeV-g2 RNA were found in the blood of infected animals, indicating that altered replication kinetics may contribute to different disease outcomes [11]. The ability of the F and G proteins to form syncytia was also reported to be reduced for HeV-g2 due to altered fusogenic capacity [12]. While the significance of syncytia in pathogenesis is unclear, it is thought to facilitate spread [13], so this difference between genotypes may contribute to altered replication and disease. However, comparative analyses of other molecular determinants of pathogenesis, such as immune evasion by the divergent phosphoprotein (P) gene products, are still lacking.

Together with the RNA genome, nucleocapsid (N) and large (L) protein, the P protein forms the ribonucleoprotein complex that is essential for viral transcription and replication [5]. The P gene also encodes the accessory proteins V and W, via mRNA editing, and C, in an alternative reading frame [14]. The best characterized functions of these proteins are in immune evasion [15,16], and roles in infection have been assessed using recombinant NiV knockouts and animal models [17,19]. Studies in ferrets and hamsters, in which NiV infection is lethal, indicated that V protein deletion produces non-lethal infections [17,19]. In contrast, W protein deletion does not prevent lethality in either animal but alters disease course in ferrets [17,19]. Roles of V and W proteins in disease are also implied by the apparent inability of Cedar virus (an Australian henipavirus that has a reduced immune evasion function and pathogenesis in animal models compared to HeV/NiV) to produce V and W [4,20].

**Fig. 1.**
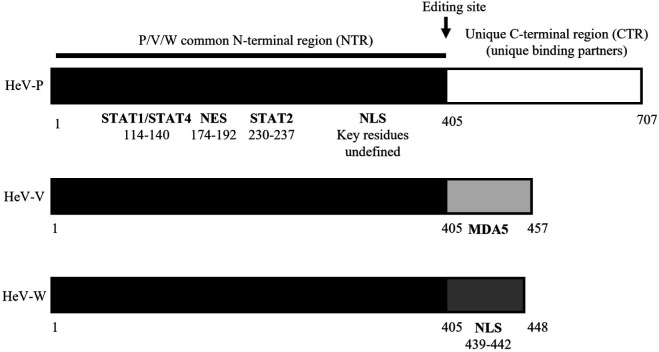
Schematic of HeV P, V and W proteins and key binding sites. The P, V and W proteins share a 405-residue NTR containing STAT1/4 and STAT2 binding sites (regions containing important residues mapped for NiV are shown) [20,25, 30, 58], a nuclear export signal (NES) [31] and a proposed nuclear localization signal (NLS; residues not mapped) [31]. Each protein has a unique CTR enabling distinct interactions, including the V protein CTR with MDA5 [27] and W protein CTR with importins (NLS residues for NiV shown) [32].

P, V and W proteins have multiple functions that depend on a common intrinsically disordered N-terminal region (NTR) and unique P, V or W C-terminal regions (CTRs) [14,21] (Fig. 1). The NTR and CTRs of P, V and W form interactions with viral and host proteins, with many of the latter known to modulate host biology, with particular roles in antagonism of antiviral responses mediated by type-I IFN cytokines [16,22]. IFN antagonism by henipaviruses and many other viruses is critical to infection [23,24], and HeV/NiV P, V, W and C proteins effect mechanisms to inhibit both the induction of IFNs following infection, and the subsequent signalling by IFN that can establish an antiviral state [15,16, 20, 25, 26]. A well-characterized mechanism is the inhibition by V protein of the pattern recognition receptor (PRR) melanoma differentiation-associated protein 5 (MDA5), which recognizes RNA virus infection leading to IFN induction [27]. As direct binding is mediated by the unique V protein CTR, P and W do not interact with MDA5 [27] (Fig. 1).

Following induction, type-I IFNs (most commonly IFNα/β) are released from cells and signal in an autocrine and paracrine fashion by binding to type-I IFN receptors. This activates signal transducers and activators of transcription (STAT) 1 and 2, which stimulate promoters containing IFN-stimulated response elements (ISREs) to activate hundreds of IFN-stimulated genes to establish an antiviral state [28]. STAT antagonism is a common viral mechanism to suppress antiviral responses [29], and the HeV P, V and W proteins bind and inhibit STAT1/STAT2 via regions in the P/V/W NTR (Fig. 1) [20,30].

Although henipavirus replication is cytoplasmic, several henipavirus proteins, including V and W, can localize to the nucleus [16,31, 32], enabling modulation of nuclear processes. Nucleocytoplasmic trafficking is important to the immune evasion functions of proteins of several viruses with cytoplasmic replication [33,35]. V and W proteins exceed the diffusion limit of the nuclear pore complex and so interact with importin α1 via their common NTR for active nuclear import, indicating that this region contains a nuclear localization signal (NLS), although the sequence comprising the NLS is yet to be defined [31]. The NTR also contains a nuclear export signal (NES) (residues 174–192) (Fig. 1) which binds exportin-1 (XPO1), such that inhibition of XPO1 using leptomycin B (LMB) increases V and W nuclear localization [31]. Notably, P protein is cytoplasmic and does not localize to the nucleus following LMB treatment, indicating that P does not undergo nuclear trafficking [31]. An additional NLS in the W protein CTR (Fig. 1) results in strong nuclear localization compared with a largely cytoplasmic localization of V [31,32]. Potential nuclear roles of V protein remain unresolved, but several have been described or predicted for W protein, including binding to inactive STAT1 in the nucleus [25] and binding the PRP19 complex, which regulates several processes, including p53 activity [36].

The HeV-g1 and HeV-g2 genomes are highly similar [HeV-g1, GenBank: AF017149.3 and HeV-g2, GenBank: MZ318101.1 (horse isolates used in this study) have 83.5% nucleotide identity]. While the N, M, F, G, L and C viral proteins have >90% amino acid identity between HeV-g1 and HeV-g2, the P, V and W proteins are more diverse (82%, 78% and 77% identity, respectively) (Table S1, available in the online Supplementary Material), consistent with the presence of large intrinsically disordered regions in the P, V and W proteins [21,37].

Given the divergence of the V protein between HeV genotypes, its central role in immune evasion and its lethality in animal infection models [17,19], we investigated whether V protein differences contribute to the distinct phenotypes observed for HeV-g1 and HeV-g2 [11]. We report that HeV-g1 virus and HeV-g1-V protein alone antagonized IFN induction more strongly than HeV-g2 virus or V protein, correlating with differences in nucleocytoplasmic trafficking. These data indicate fundamental differences in V protein function likely contributing to the differing pathogenicity of emerging HeV genotypes [11].

## Methods

### Cells and viruses

AGM kidney cells (Vero cells) and HeLa cells were maintained in Minimum Essential Medium (MEM; Gibco) supplemented with 10% FBS (CellSera Australia) and l-glutamine. HEK293T cells and *Pteropus alecto* (PaKi-T02) kidney cells [38] were maintained in Dulbecco’s Modified Eagle Medium (DMEM; Gibco) supplemented with 10% FBS (CellSera Australia) and l-glutamine. Cells were maintained at 37 °C, 5% CO_2_.

All work with infectious HeV was conducted at the Australian Centre for Disease Preparedness (ACDP) at Biosafety level 4 containment using HeV-g1 (GenBank: AF017149.3) and HeV-g2 (GenBank: MZ318101.1) isolated from horses in 1994 and 2021. Isolates were at a similar passage from the original material. Viruses were titrated by tissue culture infectious dose (TCID_50_) assay in Vero cells as described previously [39] with endpoints read at 4 days post-infection using the Reed and Muench method to find 50% endpoints by visualizing cytopathic effect [40].

### Plasmids

Constructs to express HeV-g1 and HeV-g2 P, V, W and chimeric V proteins fused to GFP were generated by PCR amplification using Q5 High-Fidelity 2x Master Mix (New England Biolabs, NEB) of viral cDNA. P genes were cloned into the pEGFP-C1 vector in frame C-terminal to GFP using the NEBuilder HiFi DNA Assembly Master Mix (NEB). To construct V and W encoding plasmids, additional guanine residues (one for V or two for W) were inserted by site-directed PCR mutagenesis using the Quik Change II kit (Agilent). V chimeras were generated using PCR of gene segments and HiFi DNA Assembly.

Constructs to express GFP-fused NiV-V-CTR, NiV-P/V/W-NTR and BeiPV were generated previously [41] along with GFP-fused rabies P [42].

### Luciferase reporter assays

For IFN/STAT1 signalling assays, HEK293T cells (1.5×10^5^ per well in 24-well plates) were co-transfected with pRL-TK (40 ng; Promega), pISRE-luc (250 ng; Stratagene) and plasmids to express GFP-fused viral proteins or controls (250 ng). After 8 h, the medium was replaced with fresh complete medium with or without IFNα (1,000 U ml^−1^; PBL Interferon Source) for a further 16 h before analysis.

For assays of MDA5-dependent IFN induction, HEK293T cells were co-transfected with pIFN-β-GL3 [250 ng; kindly provided by Lin *et al.* (McGill University) [43]], pRL-TK (40 ng), plasmids expressing human FLAG-MDA5 [125 ng; kindly provided by A.Mansell (La Trobe University)] and GFP-fused viral proteins or controls, before incubation for 24 h. Total DNA per well was kept consistent within each assay using pUC18.

All transfections were performed using FuGENE HD (Promega) according to the manufacturer’s instructions (2 µl per well in 100 µl serum free media in 24-well plates). After incubation, cells were lysed in Passive Lysis Buffer (Promega) and a dual luciferase assay whereby firefly and *Renilla* activities were quantified as previously described [41].

### Immunoblot (IB) analysis

Proteins from cell lysates were separated by SDS-PAGE (10% acrylamide) before transfer to a nitrocellulose membrane for immunoblotting with rabbit anti-GFP (Abcam, Cat #Ab6556; 1 : 5000), mouse anti-FLAG (Sigma, Cat #F1804; 1 : 1000) or mouse anti-*β*-tubulin (Sigma, Cat #T8328; 1 : 2000) followed by secondary antibodies [goat anti-rabbit HRP-conjugated (Cat #AP307P; Merck) or goat anti-mouse HRP-conjugated (Cat #AP308P; Merck)]. Visualization used ECL Lightening Plus reagent (Revvity, Cat #NEL103001EA).

### Immunoprecipitation (IP)

Cells expressing GFP-tagged viral proteins and/or FLAG-MDA5 were lysed in dilution buffer [10 mM Tris/Cl pH 7.5; 150 mM NaCl; 0.5 mM EDTA; 1× Protease Inhibitor Cocktail (Sigma-Aldrich)] with 0.5% NP-40 24 h post-transfection for 30 min at 4 °C. Co-IP was performed for FLAG followed by IB. Ten per cent of the supernatant was retained for analysis of input lysate, and the remainder was subjected to IP using anti-FLAG M2 magnetic beads (Merck) according to the manufacturer’s instructions. Beads were washed 3× with dilution buffer and resuspended in 2x SDS-PAGE sample loading buffer.

### Quantitative real-time PCR (qRT-PCR)

HEK293T cells (1.5×10^5^ per well of a 24-well plate) were mock-infected or infected with HeV-g1 or HeV-g2 at an multiplicity of infection (MOI) of 0.4, 2 or 10 for 2 h. Cell lysates were harvested for RNA extraction (MagMax-96 Viral RNA Isolation, Applied Biosystems) and qRT-PCR analysis (AgPath-ID One-Step, Applied Biosystems), following the manufacturer’s instructions. Custom primer/probe sequences were generated for IFNβ [44] and 18S rRNA (sequences obtained from CSIRO ACDP Molecular Diagnostics 18S assay, sequences available upon request).

### Confocal laser scanning microscopy (CLSM)

For live cell analysis, transfected HEK293T cells were treated with or without LMB (5.6 ng ml^−1^, 3 h) before imaging in phenol-free DMEM using a Nikon C1 inverted confocal microscope with a 60× oil immersion objective and a heated chamber. To obtain representative data to detect differences between proteins of genotypes and chimeras, we used quantitative image analysis as previously described [45,47]; specifically, we sampled >50 cells per condition before image processing and analysis using Fiji software (ImageJ) (v1.52p) to calculate the ratio of nuclear to cytoplasmic fluorescence, corrected for background fluorescence (Fn/c).

## Results and discussion

### HeV-g1-V inhibits IFN induction more potently than HeV-g2-V

As the major functions of V protein in infection appear to be in IFN antagonism [16], we compared the ability of the HeV-g1 and HeV-g2-V proteins to antagonize IFN induction and signalling. HeV and NiV are reported to antagonize IFN induction by several mechanisms [15,16], with the V protein–MDA5 interaction being particularly well-characterized and conserved across the family *Paramyxoviridae* [27,48, 49]. To assess potential genotype-specific differences, we employed a well-established IFNβ promoter-driven luciferase reporter assay [27,41]. HEK293T cells were co-transfected with plasmids for the IFNβ-luciferase assay, including a firefly IFNβ reporter and a constitutively active *Renilla* reporter for normalization, FLAG-MDA5 (which activates the IFNβ promoter when overexpressed [41]) or empty vector (pUC) and plasmids to express GFP-V proteins or GFP fused to the common NTR of NiV P/V/W (a control construct lacking the MDA5-binding CTR of V protein [27,41]).

**Fig. 2.**
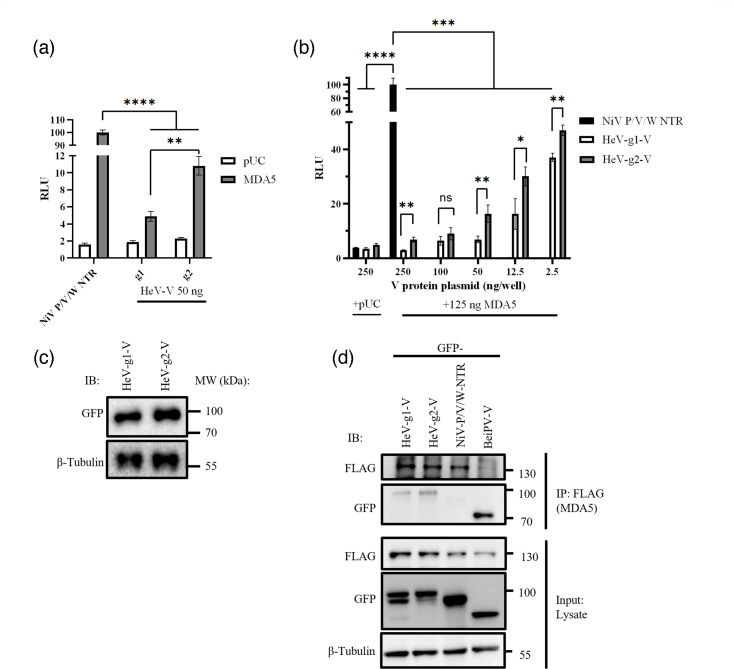
HeV-g1-V inhibits MDA5-dependent activation of the IFNβ promoter more potently than HeV-g2-V. (**a, b**) HEK293T cells were co-transfected with plasmids for an IFNβ dual luciferase assay (pIFNβ-GL3, in which firefly luciferase is controlled by an IFNβ promoter, and pRL-TK, in which *Renilla* luciferase is controlled by a constitutively active promoter [41]), plasmids to express the indicated GFP-fused viral protein and plasmid to express FLAG-MDA5 or empty vector (pUC); amounts of plasmid transfected encoding for viral proteins per well are indicated. Cell lysates were analysed for luciferase activity 24 h later; normalized RLUs (firefly/*Renilla*) are shown as a percentage of the RLU for NiV P/V/W NTR+MDA5 [mean±sd, *n*=3; data from one assay representative of three independent assays (**a**) or one assay (**b**)]. Statistical analysis used a two-tailed, unpaired t-test; *P<0.05, **P≤0.01, ***P≤0.001 and ****P≤0.0001. (**c**) IB analysis using anti-GFP and anti-*β*-tubulin on representative lysates from one of the luciferase assays conducted with 250 ng of V and in the presence of MDA5. (**d**) Cells co-transfected to express FLAG-MDA5 and the indicated GFP-fused proteins were subjected to IP for FLAG before IB analysis of input (lysate) and IP.

In cells expressing GFP-NiV P/V/W NTR, normalized luciferase activity [firefly/*Renilla*, reported as relative luciferase units (RLUs)] was clearly induced by co-expression of FLAG-MDA5 compared with pUC (Fig. 2a). MDA5-induced luciferase expression was significantly inhibited in cells expressing HeV-g1-V (Fig. 2a), consistent with previous findings that HeV-g1-V antagonizes MDA5 signalling [27]. The data also indicated conservation of this antagonistic function in HeV-g2-V, although inhibition was significantly less potent for HeV-g2-V compared with HeV-g1-V (Fig. 2a). This reduced inhibition by HeV-g2-V was observed across a range of V-protein plasmid transfection levels, with differences of up to twofold between the genotypes (Fig. 2b). IB analysis of lysates from the luciferase assays indicated similar expression levels of V protein samples (Fig. 2c). To assess the interaction of the V proteins with MDA5, we co-expressed GFP-V proteins or controls [NiV P/V/W NTR and Beilong virus (BeiPV) V protein, which efficiently binds MDA5 [41]] with FLAG-MDA5 in HEK293T cells, followed by IP for FLAG-MDA5 and IB analysis. All V proteins, but not NiV P/V/W NTR, co-precipitated with FLAG-MDA5 (Fig. 2d), with similar levels of HeV-g1 and HeV-g2-V proteins detected, indicative of comparable interaction. Taken together, these data suggest that HeV-g1-V inhibits MDA5-induced IFN induction more potently than HeV-g2-V, but this does not appear to involve substantial variation in MDA5 binding.

### HeV-g1 and HeV-g2 P, V and W proteins mediate comparable inhibition of IFN signalling

**Fig. 3.**
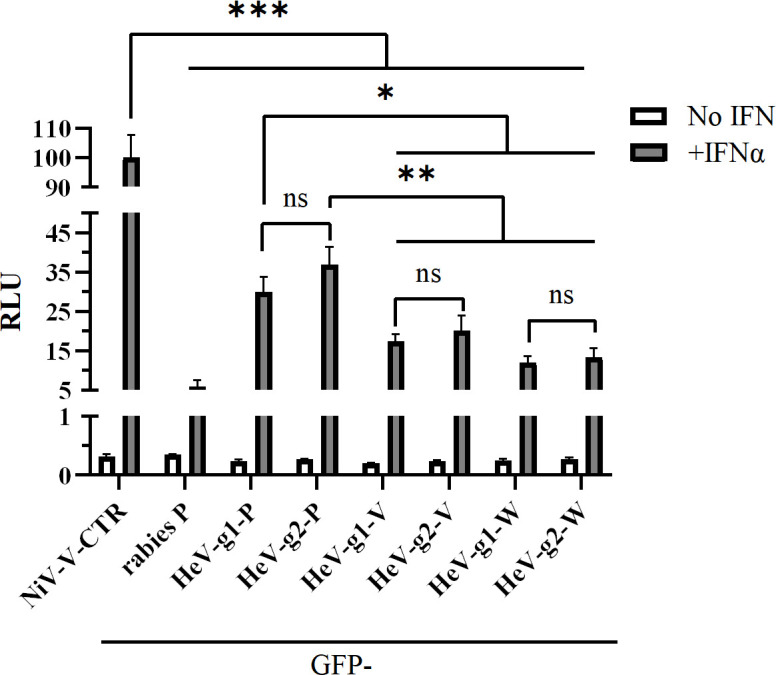
IFN/STAT1 antagonism by P, V and W proteins is conserved between HeV genotypes. HEK293T cells were co-transfected with plasmids for an ISRE-dependent dual luciferase assay (pISRE-luciferase, in which firefly luciferase is controlled by an ISRE promoter, and pRL-TK as a *Renilla* luciferase control) and plasmids to express the indicated GFP-fused proteins. At 8 h post-transfection, cells were treated with IFNα or left unstimulated, for analysis 16 h later [41]. RLUs (calculated as in Fig. 2) are shown as a percentage of the RLU obtained for NiV-M-V-CTR+IFNα samples (mean±sd, *n*=3); data are from a single assay, representative of three independent assays. Statistical analysis used a two-tailed, unpaired t-test; *P<0.05, **P≤0.01 and ***P≤0.001.

To examine whether HeV-g1 or HeV-g2-V proteins differ in antagonism of IFN signalling, we used a well-established ISRE-dependent dual luciferase gene assay [41,47, 50]. We included P and W proteins, which also inhibit IFN signalling [20,51], NiV-V-CTR (negative control lacking the STAT-binding NTR [41]) and rabies virus P protein, a potent STAT1-antagonist positive control [52,53]. IFNα treatment resulted in a strong induction of ISRE-responsive luciferase activity in cells expressing NiV-V-CTR, whereas this signalling was inhibited in cells expressing rabies P or HeV P, V and W proteins, as expected [20,30, 41, 52, 54] (Fig. 3). Consistent with data for NiV proteins [51], the HeV V and W proteins antagonized IFN signalling more potently than the P proteins. The HeV-g2 proteins inhibited IFN signalling to a similar extent as the respective HeV-g1 proteins (Fig. 3), indicating that P, V and W proteins mediate similar levels of inhibition of type-I IFN/STAT1/2 signalling. This suggests that the major difference between genotypes in IFN antagonism lies primarily in V-protein-mediated antagonism of IFN induction.

### Nucleocytoplasmic trafficking of V and W proteins differs between HeV genotypes

**Fig. 4.**
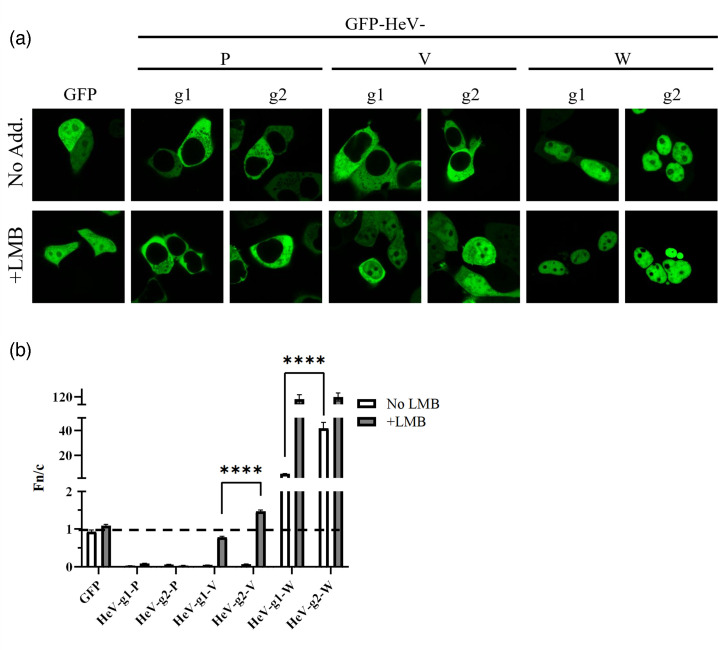
Nucleocytoplasmic localization of HeV V and W proteins differs between genotypes. (**a, b**) HEK293T cells were transfected to express the indicated GFP-HeV protein or GFP alone (~21 h), before treatment with or without LMB (3 h) and imaging of living cells by CLSM [31]. Images such as those shown in (**a**) were used to calculate the Fn/c (**b**) (mean±sem, *n*≥64 cells per condition from three independent experiments). Statistical analysis used a two-tailed, unpaired t-test; ****P≤0.0001.

As the V proteins of HeV-g1 and HeV-g2 bind MDA5 to similar extents, we considered that other V protein functions may contribute to differing antagonism of IFN induction. We thus assessed the nucleocytoplasmic localization, which has been implicated in IFN antagonist functions of other viral proteins [34,51]. GFP-P, V and W proteins were expressed in HEK293T cells before treatment with or without LMB and analysis of living cells by CLSM (Fig. 4a). As expected, HeV-g1 P and V appeared exclusively cytoplasmic, while W protein was nuclear, and LMB treatment resulted in nuclear localization of V, but not P protein [31]. Comparable results were observed for the HeV-g2 proteins, indicating conservation of trafficking mechanisms.

To quantify nucleocytoplasmic localization of the different proteins, we calculated the nuclear to cytoplasmic fluorescence ratio (Fn/c) [31]. Consistent with previous findings for HeV-g1, the Fn/c of V and W of both HeV-g1 and HeV-g2, but not P protein, increased following LMB treatment, indicating that the localization of V and W is dependent on active nuclear trafficking involving XPO1-mediated nuclear export [31] (Fig. 4b). However, the Fn/c for HeV-g2-V in LMB-treated cells, and for W in non-treated cells, was greater than that of the respective HeV-g1 proteins.

Together, these data suggest that nuclear import activity of the V protein, which is reported to involve an NLS in the NTR, may differ between HeV-g1 and HeV-g2, resulting in differing nuclear accumulation following inhibition of nuclear export. However, as it is possible that LMB does not completely inhibit XPO1 activity in these assays, we cannot exclude that differences in export may contribute. The differing localization of the W proteins in the absence of LMB treatment could be due to differing export by the NTR-localized NES, or import (involving NLSs in the NTR and CTR), or both. The lack of a difference in nuclear accumulation of the W proteins following LMB treatment (Fig. 4b) may suggest that they differ in export rather than import; however, the very high (>100) Fn/c in LMB-treated cells may preclude detection of differences in import that are apparent when export is active. Since both V and W proteins differed in trafficking, it is likely that this effect involves changes to the shared NTR, resulting in altered interactions with nuclear or cytoplasmic factors, such as the regulatory interactions of V protein with cytoplasmic MDA5 or associated cytoplasmic PRR regulators [27,55].

### Molecular mapping of HeV-g1/g2-V protein indicates that differences in MDA5 inhibition and trafficking are localized within the central region/CTR

**Fig. 5.**
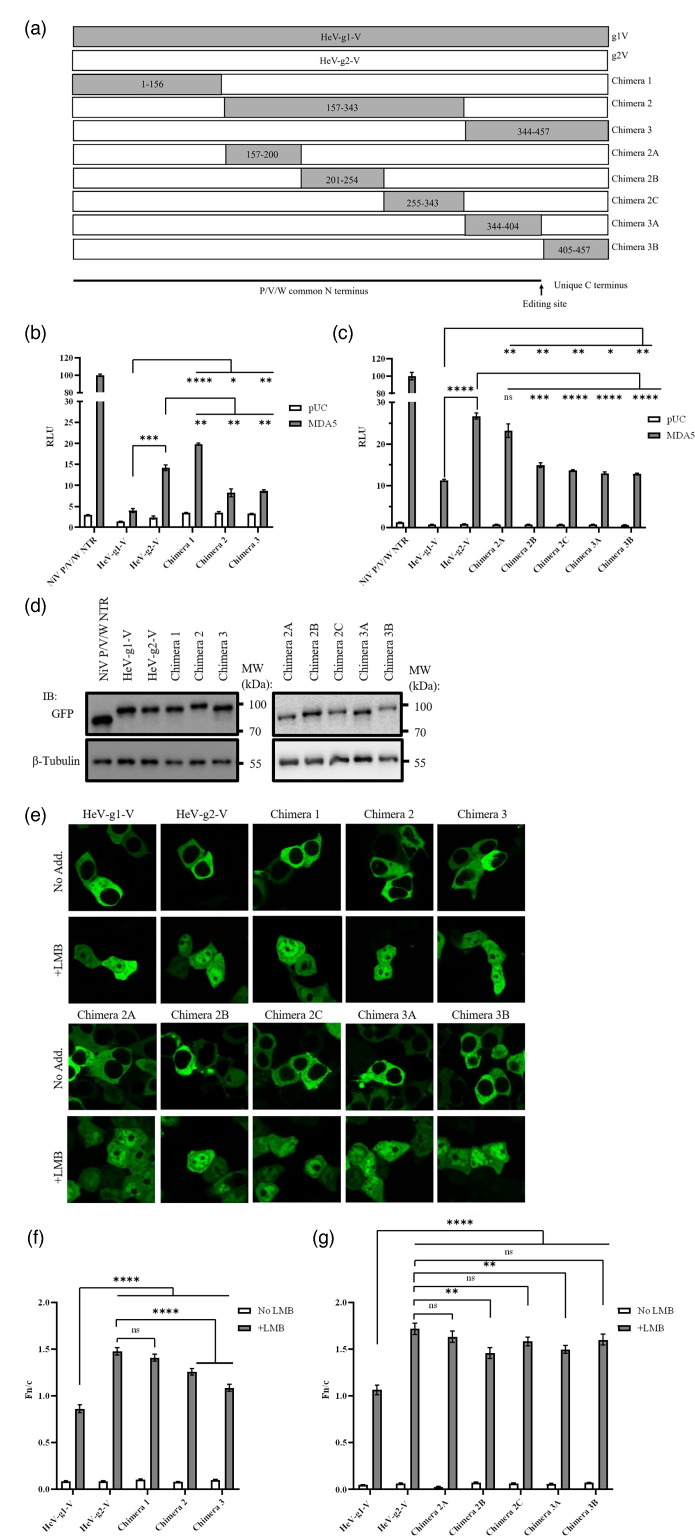
Protein chimeras differ in MDA5 antagonism and nuclear trafficking. (**a**) Schematic representation of HeV V protein chimeras generated in this study, highlighting regions substituted between HeV-g1-V and HeV-g2-V. (**b, c**) IFNβ dual luciferase assay performed as described in the legend to Fig. 2 (mean±sd, *n*=3); data from a single assay with comparable trends observed across four independent assays. (**d**) Representative IB analysis of lysates from luciferase assays. (**e–g**) CLSM analysis of nucleocytoplasmic localization, performed as described in the legend to Fig. 4 to calculate Fn/c (mean±sem, *n*≥50 cells from three separate experiments). Statistical analysis used a two-tailed, unpaired t-test; *P<0.05, **P≤0.01, ***P≤0.001 and ****P≤0.0001.

To identify the region(s) of the V proteins responsible for the differences in nucleocytoplasmic trafficking (which involves NLS/NES sequences in the NTR [31]) and MDA5 inhibition (mediated via interaction of the V protein CTR with MDA5 [27]), we generated chimeras of HeV-g1-V and HeV-g2-V proteins (Fig. 5a). The differences could result from direct, discrete changes to the specific sites/signals (which include currently undefined sequences), or indirect effects via changes to proximal/distant sequences, including conformational effects, changes to regulatory sites or changes to sites mediating other interactions. We thus initially generated three chimeras (1–3), selecting sections that would preserve known interaction sites in HeV-g1 (Figs 5a and S1) and used these to replace the corresponding regions in HeV-g2-V. Chimeras were assessed as described above to determine whether any regions of HeV-g1 protein were sufficient to enhance MDA5 antagonism and/or decrease nuclear localization of HeV-g2 (Fig. 5).

Antagonism of IFNβ induction by chimera 1 was not enhanced (and was in fact reduced) compared with HeV-g2-V, while chimeras 2 and 3 exhibited antagonistic function between that of HeV-g1-V and HeV-g2-V (Fig. 5b). This indicates that the central (residues 157–343) and C-terminal (344–457) segments, but not the N-terminal segment (1–156), of HeV-g1-V contribute to enhanced MDA5 inhibition. IB analysis of lysates from the luciferase assays confirmed similar expression of the chimeras (Fig. 5d). CLSM analysis indicated that the Fn/c for chimeras 2 and 3, but not chimera 1, was reduced compared with HeV-g2-V in LMB-treated cells (Fig. 5e, f). Thus, the central region and CTR contribute to differences in nuclear trafficking and MDA5 antagonism, consistent with a potential association of these functions.

To further dissect the regions responsible, we generated five additional chimeras replacing subsections of the central and C-terminal parts of HeV-g2-V with the corresponding sequences of HeV-g1-V (Figs 5a and S1). Chimera 2A, in which residues 157–200 containing the N-terminal NES (residues 174–192) are exchanged (Fig. 5a), did not differ from HeV-g2-V for MDA5 antagonism or nuclear trafficking, suggesting that changes in the NES or proximal sequences do not contribute to the functional differences of HeV-g1 and HeV-g2-V proteins (Fig. 5c, g).

The MDA5 antagonistic functions of chimeras 2B, 2C, 3A and 3B were strongly enhanced compared with that of HeV-g2-V, but no chimera was sufficient to recapitulate the activity of HeV-g1-V protein, indicating that several regions within residues 200–457 contribute to the differing function of the V proteins (Fig. 5c). The chimeras produced only moderate effects on nuclear trafficking, with only chimeras 2B and 3A reaching significance in reduction of Fn/c compared with HeV-g2-V (Fig. 5g). As these chimeras also impacted MDA5 antagonism, there may be an association of this function with nucleocytoplasmic trafficking of the V protein. However, the lack of a significant effect of chimeras 2C and 3B on nuclear trafficking, compared with the substantial, significant effects of these chimeras on MDA5 antagonism, does not support a simple direct relationship whereby altered nuclear import alone is responsible for reduced antagonism. Instead, these phenotypes likely reflect broader structural or conformational effects contributing to the genotypic differences between V proteins, particularly for nuclear trafficking, as the regions producing a significant effect are non-sequential. These differences may also relate to altered interactions with nuclear or cytoplasmic factors, including those involved in IFN antagonism, such that the enhanced nuclear localization of HeV-g2-V may relate to loss of cytoplasmic interactions involved in immune evasion, while increased cytoplasmic interactions for immune evasion by HeV-g1-V may result in sequestration and reduced nuclear localization.

Notably, while nuclear trafficking of the V protein is well established [31], the sequences responsible have been only partially defined, and the precise functional consequences remain unclear. By identifying regions contributing to nuclear trafficking and immune evasion, our findings provide a framework for further mechanistic analysis. Future studies defining specific sites/sequences responsible for altered trafficking and IFN antagonism may help determine the relationship between these processes and their specific contributions to pathogenesis.

### Infection by HeV-g1 induces lower levels of IFNβ than infection by HeV-g2

**Fig. 6.**
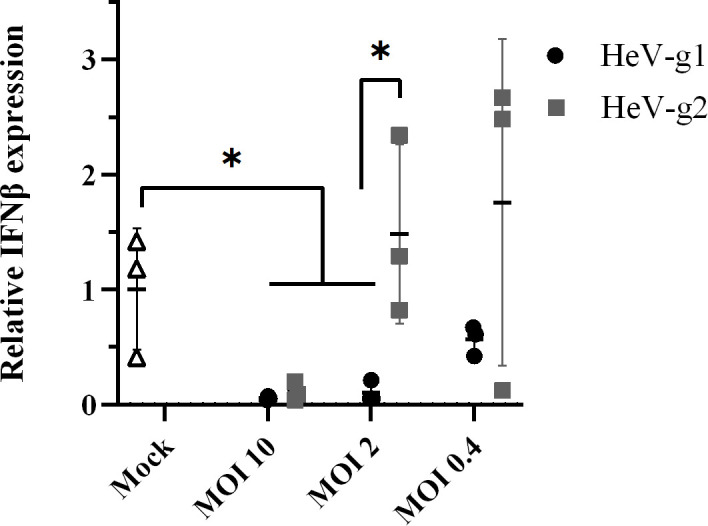
HeV-g1 infection induces lower levels of IFNβ transcripts than HeV-g2 infection. HEK293T cells were infected with HeV-g1 or HeV-g2 at the indicated MOI or were mock-infected, for 2 h before qRT-PCR analysis of IFNβ transcripts [44] and 18S rRNA. Copy numbers were calculated from standard curves generated using cells transfected with plasmids encoding IFNβ or 18S, and IFNβ values were normalized to 18S and expressed relative to the mock-infected control (mean±sd, *n*=3). Data are from a single experiment, and comparable trends were observed across three independent assays. Statistical analysis used a two-tailed, unpaired t-test; *P<0.05.

To assess if the differing phenotypes of the V proteins for IFN antagonism correlate with outcomes in infection, we infected HEK293T cells with HeV-g1 or HeV-g2 (MOI of 10, 2 or 0.4 for 2 h) and quantified IFNβ transcripts by qRT-PCR. Cells transfected to express FLAG-MDA5 or with pUC were used as positive and negative controls, confirming clear detection of MDA5-dependent induction of IFNβ transcripts (Fig. S2). In cells infected at MOI 10, little or no IFNβ transcript was detected in either HeV-g1- or HeV-g2-expressing cells, and IFNβ transcript expression was significantly reduced compared with basal transcription (mock-infected cells); these data are consistent with potent antagonism of IFNβ despite the activation of PRRs by infection (Fig. 6) [56]. At lower MOIs, IFNβ expression increased, consistent with reduced antagonism, and IFNβ transcripts showed a trend of increasing in HeV-g2-infected cells compared with HeV-g1-infected cells, with a statistically significant difference observed at an MOI of 2 (Fig. 6). Thus, it appears that HeV-g1 inhibits IFNβ production more strongly than HeV-g2 following infection, consistent with greater IFN-antagonist function of the HeV-g1-V protein (Fig. 2).

In summary, our findings show that V proteins of HeV-g1 and HeV-g2 differ in their capacity to antagonize IFN induction and that these differences correlate with differing IFN antagonism during infection by HeV-g1 and HeV-g2. The differing capacity to antagonize the antiviral IFN response is consistent with recently reported differences in viral replication in monkey, human and equine cell lines [11] (which we also observed in monkey, human and bat cell lines, Fig. S3), and more severe disease outcomes in AGMs infected by HeV-g1 compared with HeV-g2 [11]. However, as differences in viral titres were evident in Vero (monkey) cell lines [11] (Fig. S3), which are defective for IFN production [57], other factors such as altered fusogenicity of F and G protein [12] are also likely to contribute to altered replication. The relative contribution of distinct functions of V protein, F/G protein and potentially other HeV proteins on the fitness and pathogenic potential of HeV-g1 and HeV-g2 may be resolved by targeted mutagenesis and reverse genetics approaches. Overall, these data advance our understanding of the molecular determinants that distinguish emerging HeV genotypes and may contribute to differing disease risk.

## Supplementary material

10.1099/jgv.0.002256 Uncited Supplementary Material 2.

## Abbreviations

ACDP: Australian Centre for Disease Preparedness
AGMs: African green monkeys
BeiPV: Beilong virus
CLSM: confocal laser scanning microscopy
CTR: C-terminal region
DMEM: Dulbecco’s Modified Eagle Medium
F: fusion
HeV: Hendra virus
HeV-g1: HeV genotype 1
HeV-g2: HeV genotype 2
IB: immunoblot
IFN: interferon
IP: immunoprecipitation
ISREs: IFN-stimulated response elements
L: large
LMB: leptomycin B
MDA5: melanoma differentiation-associated protein 5
MEM: Minimum Essential Medium
MOI: multiplicity of infection
N: nucleocapsid
NEB: New England Biolabs
NES: nuclear export signal
NiV: Nipah virus
NLS: nuclear localization signal
NTR: N-terminal region
P: phosphoprotein
PRR: pattern recognition receptor
qRT-PCR: quantitative real-time PCR
RLUs: relative luciferase units
STAT: signal transducers and activators of transcription
TCID_50_: tissue culture infectious dose
XPO1: exportin-1
