# Supplementary material for: Hendra virus genotypes 1 and 2 differ in V protein-mediated immune evasion
Source: J Gen Virol. 2026 Apr 21;107(4):002256. doi: 10.1099/jgv.0.002256 (PMC13098992; doi:10.1099/jgv.0.002256)
Supplement: Uncited Supplementary Material 2. [file jgv-107-02256-s001.pdf]

Supplementary figures and tables

Hendra virus genotype 1 and 2 differ in V protein-mediated immune evasion

<u>Table 1</u> Comparison of protein identity between HeV g1 (GenBank: AF017149.3) and HeV-g2 (GenBank: MZ318101.1), using Geneious software (version 2020.2.5).

**Protein**		**Length (amino acids)**	**% Identity**
**N**		532	96.6
**M**		352	95.7
**F**		546	95.4
**G**		604 (genotype 1)	92.5
		603 (genotype 2)	
**L**		2244	95.7
**P**	Full length	707	82.3
	Common N-terminus	405	76.7
	Unique C-terminus	302	89.7
**V**	Full length	457	77.7
	Unique C-terminus	85	84.3
**W**	Full length	448	77.2
	Unique C-terminus	43	81.4
**C**		166	94

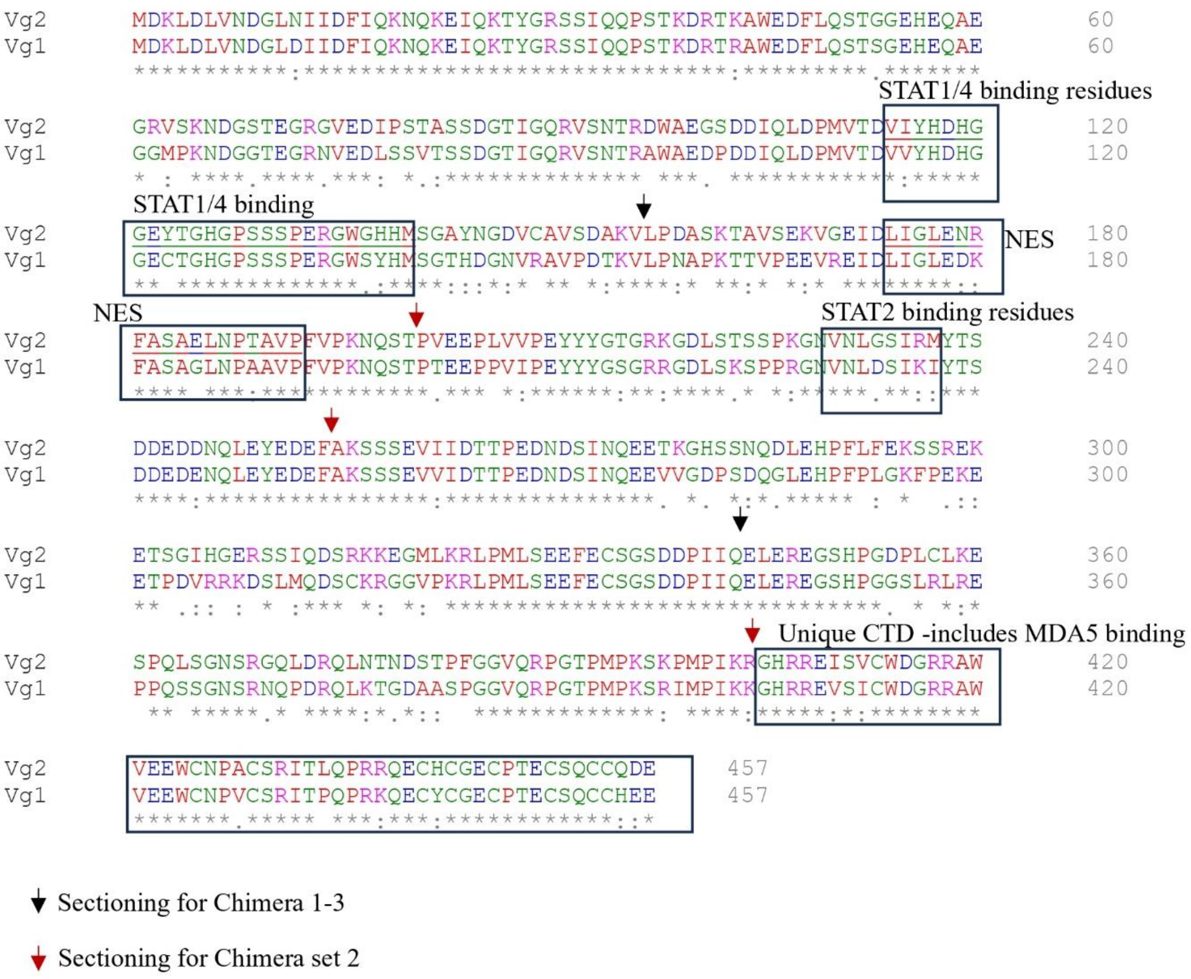

<u>Supplementary Figure 1</u>Alignment of HeV-g1 (GenBank AF017149.3) and HeV-g2 (GenBank MZ318101.1) V proteins. Sequences were aligned using Clustal Omega (conserved residues (*) and similar residues (:)shown); known interaction domains/regions for STATs, MDA5 and XPO1 (NES), and the unique CTR are indicated. Arrows indicate borders of regions exchanged in V protein chimeras (summarised in Figure 6). Font colour indicates: negatively charged (blue); positively charged (pink); polar (green); hydrophobic (red) residues.

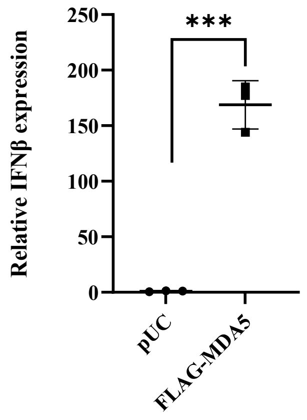

<u>Supplementary Figure 2</u> **IFNβ transcripts in HEK293T cells are stimulated by FLAG-MDA5**. HEK293T cells were transfected with plasmids to express FLAG-MDA5 or pUC for 24 h before qRT-PCR analysis of IFNβ transcripts (56) and 18S rRNA. Copy numbers were calculated from standard curves generated using cells transfected with plasmids encoding IFNβ or 18S, and IFNβ values were normalised to 18S and expressed relative to pUC control (mean ± SD, n = 3). Data are from a single assay, representative of four independent assays. Statistical analysis used a two tailed, unpaired t-test; ***$P \leq 0.001$.

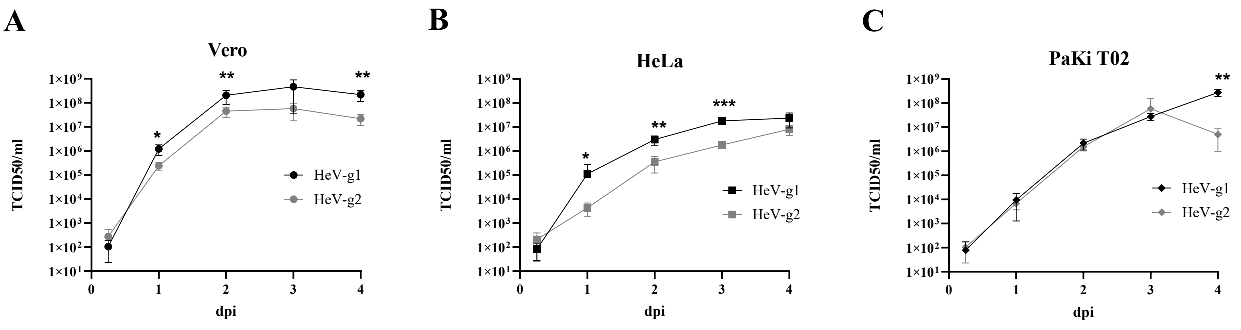

<u>Supplementary Figure 3</u> HeV-g1 reaches higher titres than HeV-g2. (A)Vero, (B) HeLa and (C) PaKi T02 cell lines were infected with HeV-g1 or HeV-g2 at a multiplicity of infection (MOI) of 0.01 before $TCID_{50}$ analysis of supernatants at 6 h and 1, 2, 3 and 4 days post infection (dpi) (mean ± SD, n = 4). Statistical analysis used a two tailed, unpaired t-test; *P<0.05, **P≤0.01, ***P ≤0.001.
